# Malignant Disease of the Uterus*Author’s abstract of a paper read before the Kentucky State Medical Society.

**Published:** 1898-07

**Authors:** Louis Frank

**Affiliations:** Louisville, Ky.


ATLANTA

### Medical and Surgical Journal.

VOL. XV. JULY, 1898. No. 5.

L. B. GRANDY, M.D., DUNBAR ROY, M.D., EDITORS.

M. B. HUTCHINS, M.D., BUSINESS MANAGER.

### ORIGINAL COMMUNICATIONS.

### MALIGNANT DISEASE OF THE UTERUS.*

BY LOUIS FRANK, M.D.,

LOUISVILLE, KY.

The frequency of malignant disease of the uterus; that it most often occurs during the time of life when symptoms resulting therefrom are apt to be normally present; the frequency of recurrences after radical operations; that many of these patients are not seen until too late for operative procedures, with other equally important features, make the subject one which the gynecologist has given most serious consideration.

Uterine cancer occurs most often between the ages of thirty-five to forty and from fifty to sixty, though it may develop at any age after the female reaches womanhood. It is found in every grade of life, and in nulliparæ as well as multiparæ; in the latter, however, it occurs more frequently than in the former, being comparatively rare in the nulliparous. It is less frequent in wealthier classes, even in women who have borne children, because they are

*Author's abstract of a paper read before the Kentucky State Medical Society.

better able to observe precautions which may obviate factors sometimes acting as predisposing causes.

We recognize three forms of cervical cancer, viz.: (1) The cauliflower or papillomatous, (2) the nodular or parenchymatous, and (3) the ulcerative or excavating—the *Carcinoma mucosa colli* of some authors. Pozzi also describes a form beginning at the posterior junction of the vagina and cervix, which he terms *carcinoma liminare.*

Of most importance is early recognition of the disease; therefore we should know its early symptoms and clinical course. It is also well to bear in mind structures which may be affected secondarily. The bladder or rectum may become involved, giving rise to vesical and rectal symptoms; we may also have nodules formed in the peritoneum. Cases so extensively diseased are necessarily inoperble, therefore necessarily fatal. Lymphatic involvement occurs earlier than has ordinarily been supposed, and because of this fact there are few permanent cures following radical operations for uterine cancer. We have both superficial and deep pelvic lymphatics, which may early become the seat of carcinomatous infiltration, so that recurrence is rapid after the organ has been removed. Involvement of the lymphatics may be so slight that its detection is impossible by bimanual examination, just as in cancer of the breast the axillary glands may be involved without exhibiting an appearance indicative thereof.

The earlier symptoms of cancer are most important. Among the earlier is prolongation of the menstrual flow, or the occurrence of metrorrhagia. Menorrhagia may be so slight that cases are neglected during the period when most could be done for them. When we remember symptoms attending the menopause, and the time malignant disease usually develops, one will not wonder why women believe all manifestations occurring at that period to be normal; and even the physician who has been consulted often lightly dismisses the case with the statement that "this is due to the change of life," not examining the patient to ascertain whether there be a local cause for the hemorrhage. This is certainly a mistake, and I would impress upon you that it is necessary to examine every woman suffering from menorrhagia or metrorrhagia during the climacteric. All cases are not due to cancer, but many are.

A suspicious symptom is the occurrence of leucorrhea, which may be slightly odorous, between the menstrual epochs, and the latter may or may not be accompanied by pain. Odorous discharge is a valuable sign, though it may not occur until after recognition of the cause thereof will be of no value.

In so-called parenchymatous or nodular cancer pain may be the first and most prominent symptom, which is also true of cancer of the body of the uterus. Pains—pricking, lancinating and cutting, radiating throughout the pelvis, down the hips and up through the abdomen—are valuable diagnostic signs. They may be so slight at first as to create no suspicion or alarm, but when accompanied with hemorrhage or odorous discharge, we may be positive that ulceration and necrosis are going on. There may be little or no loss of flesh; the appetite may be slightly or not at all impaired; there may be no cachexia—nothing upon which to base the diagnosis except slight hemorrhage, with the revelations made by the examining finger and the microscope. With loss of flesh, and cachexia or cancerous toxemia, the disease has reached a point where radical treatment will probably be of no value.

Upon digital examination in cases where the vagina and a portion of the cervix are involved, it is usually an easy matter to determine the nature of the trouble.

The most difficult to diagnose are early cases of cancer of the cervix and those where, without cervical disease, there is cancer of the body. We may think our patient has only an endometritis, or from the history that she has had an incomplete abortion; we curette her, the hemorrhage returns after a short time, and then are we first suspicious of the true nature of the trouble.

Menorrhagia or metrorrhagia, no matter how slight, nor when in the life of the woman they occur, even in the absence of other symptoms discoverable by vaginal palpation or from the history, should be an indication for microscopical examination of scrapings from the uterus. It is well, in cases with slight nodulation about the cervix, in which doubt exists, to excise a portion and examine microscopically. In one of the two cases in which I have operated without recurrence the diagnosis was made by the microscope. The microscope, however, does not always give entirely satisfactory results. In the variety of cancer having its origin in glandular structure, the resemblance to the mucosa, and especially one which has been the seat of long-continued inflammation, is so striking as to render a positive opinion almost impossible. There is a variety of cancer of the uterus which begins, I believe, as an adenoma, and which might be better termed "malignant adenoma" than cancer, where even the microscopist may be unable to say positively whether the growth is malignant or not. In such cases I would trust rather to the opinion of the clinician than the microscopist. With the aid of the microscope it may be difficult to differentiate between carcinoma of the body of the uterus and a fibroid tumor. Still, carefully weighing facts elicited by close questioning, taking into consideration every point in the clinical history, with the probability of malignancy from the microscopist's report, we should be able to arrive at a positive conclusion.

Having made the diagnosis, what is to be done? If there is a chance of removing the diseased structures, total extirpation should be undertaken. Even with the involvement of the pelvic lymphatics, permanent cures may result by removal of the uterus with the lymphatics, through the abdomen, as has been done by Clark of the Johns Hopkins Hospital. His work has shown how early there may be involvement of the lymphatics, which, as previously stated, is often the cause of recurrence when all disease has apparently been removed. Where there is involvement of the vagina, if there is fixation of the organ, if there is cachexia, nothing is to be gained by radical operation, and I believe we are not justified in subjecting the patients to the dangers of an operation of this magnitude. Curettage will lessen the hemorrhage, and curettage with cauterization will render the patients more comfortable, cause a cessation of the discharge, and prolong life even more than extirpation of the organ. My experience is that after extirpation, which I have done in several instances, recurrence is prompt, and the disease has, if anything, run a more rapid course than had I resorted to the other treatment.

We should impress upon patients, and surgeons should impress upon general practitioners, that every woman complaining of pelvic trouble, any irregularity of the menses, any copiousness of the discharge, either at the menstrual period or other times, should be subjected to examination by a competent diagnostician. Women, knowing little about their generative organs, hardly appreciate the importance of grave and never slight symptoms, and this, in connection with their natural modesty, often prevents discovery of lesions which, having been recognized in time, would have enabled us to effect cures.

TO SUMMARIZE.

(1) Cancer may occur at any time after the beginning of the menstrual life of the woman.

(2) The early symptoms are oftentimes obscure.

(3) The least irregularity during the climacteric should arouse our suspicions.

(4) Suspicious cases should be subjected to microscopic examinations.

(5) Early operation is the only hope for cure.

(6) Extirpation, after the disease is very evident, after appearance of cachexia, is harmful rather than beneficial. In these cases our efforts should be directed towards making the patient more comfortable.

(7) Finally, it is our duty to insist upon women consulting the physician for any irregularities in the menstrual flow.

